# Risk Evaluation of Azithromycin-Induced QT Prolongation in Real-World Practice

**DOI:** 10.1155/2018/1574806

**Published:** 2018-10-14

**Authors:** Young Choi, Hong-Seok Lim, Dahee Chung, Jung-gu Choi, Dukyong Yoon

**Affiliations:** ^1^Department of Biomedical Informatics, Ajou University School of Medicine, Suwon, Gyeonggi-do, Republic of Korea; ^2^Department of Biomedical Sciences, Ajou University Graduate School of Medicine, Suwon, Gyeonggi-do, Republic of Korea; ^3^Department of Cardiology, Ajou University School of Medicine, Suwon, Republic of Korea

## Abstract

**Background:**

Azithromycin exposure has been reported to increase the risk of QT prolongation and cardiovascular death. However, findings on the association between azithromycin and cardiovascular death are controversial, and azithromycin is still used in actual practice. Additionally, quantitative assessments of risk have not been performed, including the risk of QT prolongation when patients are exposed to azithromycin in a real-world clinical setting. Therefore, in this study, we aimed to evaluate the risk of exposure to azithromycin on QT prolongation in a real-world clinical setting using a 21-year medical history database of a tertiary medical institution.

**Methods:**

We analyzed the electrocardiogram results and relevant electronic health records of 402,607 subjects in a tertiary teaching hospital in Korea from 1996 to 2015. To evaluate the risk of QT prolongation of azithromycin, we conducted a case-control analysis using amoxicillin for comparison. Multiple logistic regression analysis was performed to correct for age, sex, accompanying drugs, and disease.

**Results:**

The odds ratio (OR) for QT prolongation (QTc>450 ms in male and >460 ms in female) on azithromycin exposure was 1.40 (95% confidence interval [CI], 1.23-1.59), and the OR for severe QT prolongation (QTc>500 ms) was 1.43 (95% CI, 1.13-1.82). On the other hand, the ORs on exposure to amoxicillin were 1.06 (95% CI, 0.97-1.15) and 0.88 (95% CI, 0.70-1.09). In a subgroup analysis, the risk of QT prolongation in patients aged between 60 and 80 years was significantly higher when they are exposed to azithromycin.

**Conclusions:**

The risk of QT prolongation was increased when patients, particularly the elderly aged 60-79 years, were exposed to azithromycin. Therefore, clinicians should pay exercise caution using azithromycin or consider using other antibiotics, such as amoxicillin, instead of azithromycin.

## 1. Introduction

Azithromycin is a macrolide antibiotic that is widely used to treat various infectious diseases such as respiratory and urinary tract infections. Several studies have reported the association between azithromycin and QT prolongation [1–3]. Consequently, the US Food and Drug Administration has added a warning to the drug insert. As QT prolongation can lead to a life-threatening arrhythmia, known as torsade de pointes, the drugs that cause QT prolongation have strictly been controlled. In a previous study, torsade de pointes was reported in about 1% of patients with QT prolongation after exposure to azithromycin [4]. The risk factors for torsade de pointes are female sex, older age, heart disease, exposure to other QT interval prolonging drugs or metabolic inhibitors, hypokalemia, and bradycardia [5]. Associations between azithromycin and other serious arrhythmias (e.g., ventricular tachycardia, ventricular fibrillation, ventricular flutter, or cardiac arrest) have also been reported [6].

However, many conflicting results suggesting that azithromycin exposure does not concern cardiovascular events have been reported [7–13]. QT prolongation was primarily observed only in patients with a high baseline risk (e.g., preexisting cardiovascular conditions or concomitant use of other QT-prolonging drugs) [13]. In another study, no significant association was found between azithromycin and cardiovascular death in young and middle-aged adults [10]. Despite the conflicting results, azithromycin is still largely used in real-world clinical settings [14]. Although the risks of QT prolongation and cardiovascular events need to be considered, azithromycin is recommended because it is highly effective for infection control and has relatively few other side effects [1, 15, 16].

Most of the existing studies were conducted in a small dataset or were meta-analyses reanalyzing the results from other studies. Although associations have been suggested, quantitative assessments of risk, including the risk of QT prolongation, when patients are exposed to azithromycin have not been performed in a real-world clinical setting.

Therefore, the aim of this study was to evaluate the risk and risk factors of QT prolongation after exposure to azithromycin using a 21-year medical record database and electrocardiogram (ECG) test results from a tertiary teaching hospital. First, we assessed the odds ratio (OR) of azithromycin use in patients who experienced QT prolongation in a real-world clinical setting. Second, subgroup analysis was performed to determine risk factors causing QT prolongation on azithromycin exposure.

## 2. Methods

This retrospective study was conducted based on electronic health records (EHRs). The Institutional Review Board of Ajou University Hospital approved (IRB No. AJIRB-MED-MDB-18-101) and waived the requirement for informed consent.

### 2.1. Data Resources

We used the EHR database of Ajou University Hospital, a tertiary teaching hospital in Korea, between January 1996 and May 2015. The database included 139,414,316 prescriptions, 290,926,544 laboratory test results, and 2,621,773 patient demographics.

QTc values from the same observational period were extracted from the local ECG repository in the MUSE™ system. The ECG report typically contains both alphanumeric values and waveform graphs. The QTc data were extracted by parsing alphanumeric data from the PDF data extracted from the ECG repository by web-scraping technique [17].

### 2.2. Study Design and Population

**Figure 1:**
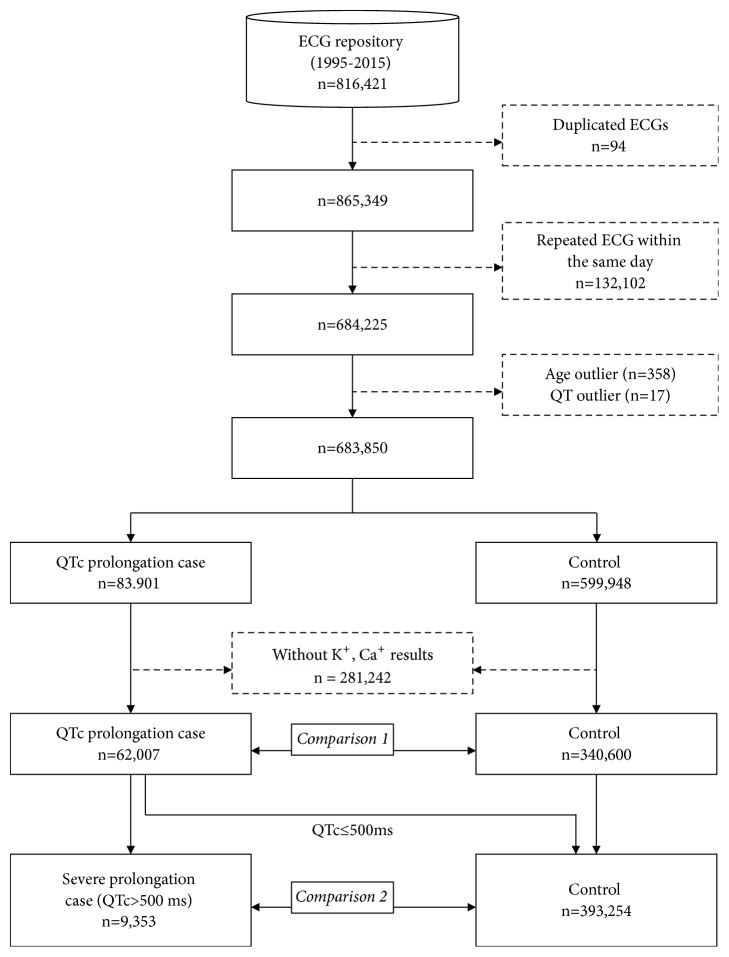
*Overview of the study process.* Among the 816,421 ECG results, 402,607 ECGs which have no duplicated or repeated measurements, no outlier, and potassium (K^+^) and calcium (Ca^+^) results within one-year before ECG measurement were enrolled and divided into QT prolongation case (n=62,007) vs. control (n=340,600) or severe QT prolongation case (n=9,353) vs. control (n=393,254).

We designed a case-control study to compare variations in use of medications, comorbidities, and laboratory test results among patients who had QT prolongation to those who did not (Figure 1). QT prolongation was defined as QTc (calculated using the Bazett formula) greater than 450 ms for males and 460 ms for females [18]. Severe QT prolongation was defined as QTc>500 ms in both males and females.

With regard to the selection of study participants, we identified 816,421 subjects who underwent ECG from January 1996 to May 2015. We excluded those with the following that rendered ECG or EHR information unclear: (1) duplicated dates of ECG measurement (n=94); (2) two or more consecutive ECG measurements within a day because of the possibility of obtaining an abnormal value several times to fit the normal or the possibility of reversing the lead for other clinical reasons (n=132,102); (3) unclear age (n=358); and (4) zero QT value (n=17). Of the 683,850 subjects, we identified 83,901 QT prolongation cases and 599,948 controls. Additionally, we excluded those without laboratory test data such as serum potassium and calcium levels within 1 year before the ECG examination date. Overall, 62,007 QT prolongation cases and 340,600 controls were enrolled (comparison 1). To evaluate the association with severe QT prolongation, 9,353 cases and 393,254 controls were enrolled (comparison 2).

### 2.3. Definition of Covariates

We assessed known or potential factors for the risk of QT prolongation. Demographic factors included sex and age at the ECG examination date. Comorbidities were included by reviewing medical records a year before the ECG measurement date based on the International Classification Disease-10. These comorbidities were myocardial infarction, congestive heart failure, ischemic stroke, hemorrhagic stroke, diabetes mellitus, hypothyroidism, renal disease, AIDS/HIV, alcohol abuse, drug abuse, liver disease, and severe liver disease. For laboratory data, we included the latest serum potassium and calcium levels within a year before the ECG measurement date. Based on previous studies, we included the use of medications known to affect QT prolongation. We identified whether there was any exposure of the medications within 7 days before the ECG measurement date. The types of medications and comorbidities are shown in Tables S1 and S2.

### 2.4. Statistical Analysis

We calculated the distribution of the patient characteristics, laboratory test results, comorbidity, and medication use for the QT prolongation cases and controls from 1995 to 2015. Pearson's chi-square test and t-test were conducted to compare between the QT prolongation cases and controls. We performed logistic regression to evaluate the relationships between azithromycin and QT prolongation. A *p* value <0.05 was considered statistically significant. Data management was performed using Microsoft SQL Server (Microsoft Corp), and all statistical analyses were conducted using the SAS software package (ver. 9.4; SAS Institute, Cary, NC, USA).

## 3. Results

### 3.1. Baseline Patient Characteristics

Tables 1 and S3 show baseline characteristics of the QT prolongation and control groups. The total number of case-control subjects was 402,607. Of these subjects, 62,007 (15.4%) and 340,600 (84.60%) belonged to the QT prolongation and control groups, respectively. The proportion of patients using azithromycin within 7 days before the ECG measurement date in the QT prolongation group was higher than that in the control group (0.65% vs. 0.27%), whereas the proportion of subjects using amoxicillin in the QT prolongation group (1.18%) was lower than that in the control group (1.39%). The proportion of QT prolongation was higher for subjects who were male and older and for those with lower potassium and calcium levels. In the analysis of severe QT prolongation, the same trend was observed, but the proportion of QT prolongation was higher in females (Table S4).

### 3.2. Comparison of QT Prolongation Risk between Exposure to Azithromycin and Amoxicillin

Table 2 shows the results of multiple regression analysis, which assessed QTc prolongation associated with the use of azithromycin and amoxicillin. After adjusting for the potential or known risk factors for QT prolongation, the use of azithromycin was more likely to be associated with QT prolongation (OR [95% CI], 1.40 [1.23-1.59]), but the use of amoxicillin was not associated with QT prolongation (OR [95% CI], 1.06 [0.97-1.15]). The OR of azithromycin for severe QT prolongation was 1.43 (95% CI, 1.13-1.82).

### 3.3. Other Risk Factors Associated with QT Prolongation

Higher odds for QT prolongation were associated with being male and older and having cardiovascular diseases, such as myocardial infarction, congestive heart failure, ischemic stroke, and hemorrhagic stroke (Table 2). QT prolongation was associated with taking medications within 7 days before the ECG measurement date. These medications included antiarrhythmic drugs, fluoroquinolone antibiotics, macrolide antibiotics, antihistamines, anesthetic/sedative, bronchodilators (beta-agonists), gastrointestinal promotility, gonadotropin-releasing hormone agonists and antagonists, antipsychotics, and vasodilator drugs. By contrast, females showed higher OR for severe QT prolongation (Table 2).

### 3.4. Risk Factors for QT Prolongation on Azithromycin Exposure

**Figure 2:**
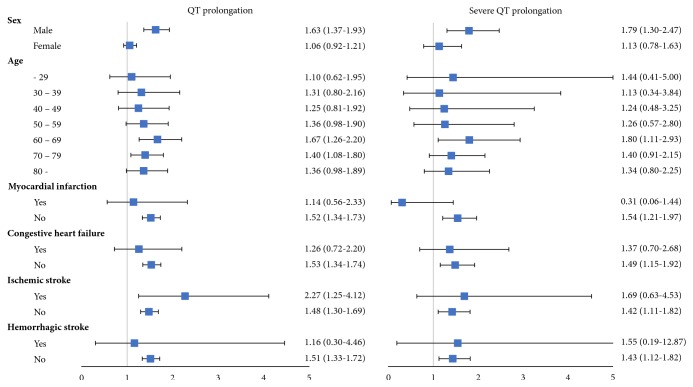
*Subgroup analysis for evaluation of risk factors for QT prolongation when azithromycin is prescribed.* Odds ratio was increased in elderly (60-79 years) and male patients in both QT prolongation and severe QT prolongation.

Figure 2 shows the subgroup analysis for association between azithromycin and QT prolongation according to sex, age, and cardiovascular disease. Azithromycin use among males was more likely to be associated with QT prolongation (OR [95% CI], 1.63 [1.37-1.93]). With regard to age group, azithromycin use in patients aged 60-69 and 70-79 years was significantly associated with a higher risk of QT prolongation (OR [95% CI], 1.66 [1.26-2.19] and 1.40 [1.09-1.81], respectively). Results for severe QT prolongation also showed a similar pattern. The OR for severe QT prolongation induced by azithromycin was higher in males and 60-69-year-old patients.

## 4. Discussion

The study findings revealed that the risk of QT prolongation with regard to azithromycin exposure was higher in males and the elderly group (ages 60-79 years).

Our findings were consistent with those of previous studies. The relationship between azithromycin and QT prolongation was statistically significant [1, 2]. However, some recently published studies have shown conflicting results with previous findings and suggested that risk is not high in all patients, but only in elderly patients or patients with cardiovascular disease [10, 13]. This trend was also observed in our study. Figure 2 shows that increasing age increased QT risk with azithromycin exposure, and the risk of QT prolongation was relatively higher in patients with ischemic stroke. In other words, our findings are not only consistent with that of a study that suggested the relationship between azithromycin and QT prolongation, but also explain the contradictory results between recent studies and earlier ones.

We also obtained results that are consistent with previous reports on amoxicillin. Amoxicillin has been used for comparison with azithromycin in many studies because it is known to have similar indications to QT prolongation [19]. Previous studies reported that amoxicillin is not associated with QT prolongation or cardiovascular death, and the OR for amoxicillin in this study was 1.06 (95% CI, 0.97-1.15), which supported the lack of association with QT prolongation.

Other covariates known to be associated with QT prolongation mostly showed significant relationship (Table 2). For example, exposure to QT prolongation-prone drug (antiarrhythmics, antipsychotics, etc.), myocardial infarction, congestive heart failure, cerebrovascular disease, liver disease, and renal disease were associated with increased risk of QT prolongation. Furthermore, lower serum levels of potassium and calcium were associated with increased risk of QT prolongation, with odds ratios of 0.68 (0.67-0.69) and 0.65 (0.64-0.65), respectively [20, 21].

The OR for QT prolongation after azithromycin exposure in the entire population was 1.4 (1.23–1.59). In a subgroup analysis, the risk was not increased by azithromycin (OR=0.98 [0.56-1.72]) at ages below 29 years. However, as the age increased, the risk increased gradually. The risk was statistically significant in patients aged 60-69 (OR [95% CI], 1.66 [1.26-2.19]) and 70-79 years (1.40 [1.09-1.81]). However, the risk tended to decrease again from the age of 70 years, and no statistical significance was found again after the age of 80 years.

In this study, the risk of QT prolongation in men was higher (Table 2). QT length in females is known to be relatively longer than that in males [22]. Therefore, according to the definition of the 2009 American Heart Association/American College of Cardiology Foundation/Heart Rhythm Society recommendations [18], we defined QT prolongation as QTc>450 ms for males and QTc>460 for females. In other words, the difference in QT length according to sex was already adjusted in the definition of QT prolongation. Another study, which analyzed a general population aged 55 years and older, also classified QT prolongation differentially over sex (QTc>450 for males and QTc>470 for females) [23]. In the study, the proportion of males was higher (57.6% vs. 42.4%) in the QT prolongation group and lower in the normal QT group (35.1% vs. 64.9%), as our results demonstrated. However, the definition of severe QT prolongation was used, and the OR in females was higher. Notably, the OR for severe QT prolongation was higher in females (Table 2), but in subgroup analysis, male sex seemed to be more affected by azithromycin exposure (Figure 2).

This study can be used to estimate the minimal incidence of QT prolongation events. QT prolongation was observed in 402 out of 1,305 individuals who were prescribed azithromycin and underwent ECG within 7 days (Table S3). At least 30.8% of patients had experienced QT prolongation. However, in patients exposed to azithromycin, it is not possible to confirm that the proportion of QT prolongation was higher in those who did not undergo ECG. Moreover, patients with normal ECG findings may have had QT prolongation at another time, because only one ECG result was included in the analysis.

Therefore, it might be helpful to conduct ECG monitoring during azithromycin use, especially in elderly patients, to prevent QT prolongation-related critical events. Nowadays, there are many trials to monitor ECG in patients' daily life [24, 25]. This approach could be useful for people who need regular ECG monitoring.

The significance of this study's findings lies in the quantitative data obtained on assessing the relationship between azithromycin and QT prolongation using large-scale ECG findings. Furthermore, using linked EHR data, we could include and adjust diverse covariates that could have an effect on QT interval such as diseases, drug exposure, and metabolic abnormalities (potassium and calcium). Currently, we have collected all ECG waveforms measured in the intensive care unit [26]. In contrast to ECGs conducted in a general ward, we could gather ECGs of all periods of hospitalization in an intensive care unit. We plan to conduct the same analysis but based on a cohort study design using ECGs gathered from intensive care unit monitoring devices in our future study. However, using those data for analyzing QT interval requires an accurate tool to measure QT interval in real time.

This study has some limitations. First, the ECG measurement itself might not be a general clinical situation. In this study, the reason for ECG measurement was not investigated. Therefore, our finding might not represent the whole population and the results may be biased. This factor should be considered when interpreting the results of this study. Second, ECG measurements were obtained using different machines with different versions of algorithms over 20 years. The hospital used mac 1200, mac 5500, and mac 5500 HD ECG, which were produced by GE Healthcare. The algorithm versions in each machine were different (12SL versions 7, 13, 22). However, all ECGs were obtained using GE Healthcare ECG machines. Because all of them were approved by the US Food and Drug Administration, the quality of the algorithms might have been controlled well. Third, this study was conducted based on a case-control design. Because the ECG results were not obtained constantly in a general ward and only provided information at the time when ECGs were conducted, a cross-sectional approach was performed.

## 5. Conclusion

QT prolongation risk was increased by azithromycin in real-world clinical practice. The clinical implication of our study is that we quantitatively evaluated the risk of QT prolongation induced by azithromycin using EHR data of a tertiary teaching hospital. In particular, higher risk was observed in the elderly group (aged 60 to 80 years). Therefore, caution should be exercised when using azithromycin for elderly patients, or amoxicillin may be a better treatment alternative for them.

## Figures and Tables

**Table 1:** Baseline characteristics of the subjects.

	QT prolongation			Severe QT prolongation		
	Case	Control	p-value	Case	Control	p-value
Total	62007 (15.4)	340600 (84.6)		9353 (2.3)	393254 (97.7)	
Sex			<.0001			<.0001
Men, n (%)	33112 (53.4)	174630 (51.3)		4519 (48.3)	203223 (51.7)	
Women, n (%)	28895 (46.6)	165970 (48.7)		4834 (51.7)	190031 (48.3)	
Age, Mean (SD)	58.0 (18.3)	47.9 (19.7)	<.0001	61.2 (17.5)	49.2 (19.8)	<.0001
Age, n (%)			<.0001			<.0001
-29	4693 (7.6)	62653 (18.4)		513 (5.5)	66833 (17)	
30-39	4468 (7.2)	46430 (13.6)		536 (5.7)	50362 (12.8)	
40-49	8244 (13.3)	60832 (17.9)		1092 (11.7)	67984 (17.3)	
50-59	11593 (18.7)	63102 (18.5)		1522 (16.3)	73173 (18.6)	
60-69	14088 (22.7)	58161 (17.1)		2126 (22.7)	70123 (17.8)	
70-79	13966 (22.5)	39668 (11.6)		2482 (26.5)	51152 (13)	
80-	4955 (8)	9754 (2.9)		1082 (11.6)	13627 (3.5)	
Potassium (mEq/l), Mean (SD)	4.1 (0.6)	4.2 (0.5)	<.0001	4.0 (0.8)	4.2 (0.5)	<.0001
Calcium (mg/dl), Mean (SD)	8.8 (0.8)	9.1 (0.6)	<.0001	8.6 (1.0)	9.1 (0.6)	<.0001
Azithromycin, n (%)	402 (0.6)	903 (0.3)	<.0001	402 (4.3)	903 (0.2)	<.0001
Amoxicillin, n (%)	733 (1.2)	4751 (1.4)	<.0001	733 (7.8)	4751 (1.2)	<.0001

**Table 2:** Results of multiple logistic regression.

	QT prolongation			Severe QT prolongation		
Variables	OR	95% CI	p-value	OR	95% CI	p-value
Azithromycin	1.395	(1.227-1.586)	<.0001	1.433	(1.127-1.822)	0.003
Amoxicillin	1.056	(0.971-1.147)	0.204	0.875	(0.703-1.089)	0.231
Male (ref. female)	1.108	(1.088-1.129)	<.0001	0.854	(0.817-0.892)	<.0001
Age (Mean/SD)	1.023	(1.022-1.023)	<.0001	1.028	(1.026-1.029)	<.0001
Potassium (mEq/l)	0.679	(0.666-0.691)	<.0001	0.537	(0.515-0.559)	<.0001
Calcium (mg/dl)	0.645	(0.636-0.654)	<.0001	0.541	(0.525-0.557)	<.0001
Antiarrhythmic drugs	3.341	(3.111-3.589)	<.0001	5.326	(4.807-5.900)	<.0001
Antianginal drugs	1.331	(0.458-3.867)	0.599			
Anticholinergic	1.132	(0.935-1.370)	0.202	0.919	(0.585-1.441)	0.712
Antimalarials	1.080	(0.933-1.251)	0.300	0.812	(0.550-1.198)	0.294
Antifungals	1.073	(0.916-1.258)	0.384	1.126	(0.816-1.552)	0.471
Fluoroquinolone antibiotics	1.198	(1.144-1.255)	<.0001	1.195	(1.083-1.318)	0.000
HIV antiretrovirals	1.048	(0.460-2.385)	0.912			
Macrolide antibiotics	1.599	(1.507-1.696)	<.0001	1.566	(1.393-1.761)	<.0001
Antihistamines	1.483	(1.304-1.687)	<.0001	1.193	(0.901-1.580)	0.219
Antineoplastic drugs	0.580	(0.527-0.638)	<.0001	0.407	(0.298-0.557)	<.0001
Anesthetic/sedative	1.151	(1.093-1.212)	<.0001	1.169	(1.039-1.314)	0.009
Opioids	1.124	(0.768-1.645)	0.549	1.016	(0.373-2.764)	0.976
Bronchodilators (beta-agonists)	1.346	(1.229-1.473)	<.0001	1.093	(0.887-1.347)	0.404
Antidiarrheals	1.091	(0.734-1.623)	0.666	1.035	(0.369-2.901)	0.948
Antiemetics	0.963	(0.870-1.066)	0.468	0.881	(0.696-1.115)	0.291
Gastrointestinal promotility	1.777	(1.729-1.827)	<.0001	1.583	(1.492-1.681)	<.0001
GnRH	1.581	(1.067-2.343)	0.023	0.612	(0.150-2.496)	0.493
Neurologic drugs	1.110	(0.895-1.376)	0.343	1.268	(0.865-1.861)	0.224
Antipsychotics	1.325	(1.259-1.395)	<.0001	1.150	(1.028-1.286)	0.014
Tricyclic and tetracyclic antidepressants	0.978	(0.898-1.065)	0.612	0.870	(0.699-1.083)	0.213
Selective serotonin reuptake inhibitors	1.119	(1.005-1.246)	0.041	1.117	(0.882-1.415)	0.358
Vasodilator drugs	1.399	(1.281-1.528)	<.0001	1.379	(1.157-1.643)	0.000
Myocardial infarction	1.306	(1.242-1.374)	<.0001	1.387	(1.257-1.530)	<.0001
Congestive heart failure	2.526	(2.403-2.654)	<.0001	3.547	(3.279-3.837)	<.0001
Ischemic stroke	1.429	(1.368-1.493)	<.0001	1.394	(1.274-1.525)	<.0001
Hemorrhagic stroke	2.276	(2.123-2.441)	<.0001	3.143	(2.797-3.531)	<.0001
Diabetes mellitus	1.167	(1.133-1.201)	<.0001	1.016	(0.950-1.086)	0.643
Hypothyroidism	0.929	(0.842-1.025)	0.140	1.033	(0.834-1.280)	0.767
Renal disease	3.119	(2.993-3.250)	<.0001	3.320	(3.077-3.582)	<.0001
AIDS/HIV	1.434	(0.893-2.301)	0.135	1.112	(0.273-4.535)	0.882
Alcohol abuse	2.108	(1.938-2.293)	<.0001	1.912	(1.645-2.221)	<.0001
Drug abuse	3.009	(2.538-3.567)	<.0001	4.356	(3.275-5.794)	<.0001
Liver disease	2.301	(2.166-2.444)	<.0001	2.349	(2.097-2.631)	<.0001
Severe liver disease	2.233	(1.991-2.505)	<.0001	1.975	(1.651-2.362)	<.0001
Year of QTc diagnosis (ref. year 1995–2000)						
2001–2005	0.965	(0.910-1.023)	0.229	1.043	(0.922-1.179)	0.507
2006–2010	1.466	(1.384-1.552)	<.0001	1.027	(0.910-1.161)	0.662
2011–2015	1.812	(1.712-1.918)	<.0001	1.149	(1.018-1.297)	0.025

GnRH: Gonadotropin-releasing hormone agonists and antagonists; AIDS: acquired immunodeficiency syndrome; HIV: human immunodeficiency virus; SD: standard deviation; OR: odds ratio; CI: confidence interval; QTc:

## Data Availability

The datasets analyzed during the current study are not publicly available due to legal restrictions imposed by the government of South Korea in relation to the Personal Information Protection Act but are available from the corresponding author on reasonable request.

## Supplementary Materials

Supplementary Materials Table S1: known or potential medications affected to QT prolongation. Table S2: comorbidities (ICD- 10 code) used in this study. Table S3: detailed baseline characteristics of the subjects in comparison 1 (QT prolongation). Table S4: detailed baseline characteristics of the subjects in comparison 2 (Severe QT prolongation).
