# Supplementary material for: Risk Evaluation of Azithromycin-Induced QT Prolongation in Real-World Practice
Source: Biomed Res Int. 2018 Oct 14;2018:1574806. doi: 10.1155/2018/1574806 (PMC6204160; doi:10.1155/2018/1574806)
Supplement: Supplementary Materials — Table S1: known or potential medications affected to QT prolongation. Table S2: comorbidities (ICD- 10 code) used in this study. Table S3: detailed baseline characteristics of the subjects in comparison 1 (QT prolongation). Table S4: detailed baseline characteristics of the subjects in comparison 2 (Severe QT prolongation). [file 1574806.f1.docx]

**Supplementary materials**

| **Table S1. Known or potential medications affected to QT prolongation** | |
| --- | --- |
| Types of medication | Ingredients |
| Antiarrhythmic | **quinidine, procainamide, disopyramide, flecainide, propafenone, amiodarone, dronedarone,** vernakalant, **sotalol,** dofetilide, ibutilide |
| Antianginal | **ranolazine, ivabradine** |
| Anticholinergic | **solifenacin, tolterodine** |
| Antimallarials | **artemether, artemether-lumefantrine, chloroquine, halofantrine, lumefantrine, delamanid, hydroxychloroquine, mefloquine, primaquine** |
| Antituberculous | Bedaquiline |
| Antifungals | **fluconazole, itraconazole, ketoconazole, posaconazole, voriconazole** |
| Fluoroquinolone antibiotics | **ciprofloxacin, gatifloxacin, levofloxacin, moxifloxacin,** ofloxacin, sparfloxacin |
| HIV antiretrovirals | **efavirenz, lopinavir-ritonavir, saquinavir, Atazanavir, nelfinavir,** rilpivirine |
| Macrolide antibiotics | **erythromycin, clarithromycin, roxithromycin, telithromycin** |
| Antihistamines | **astemizole, bilastine, hydroxyzine, terfenadine** |
| Antineoplastic drugs | **arsenic trioxide, bendamustine, capecitabine, ceritinib, cesium chloride, crizotinib, dasatinib, eribulin, fluorouracil, inotuzumab ozogamicin, nilotinib, lapatinib, lenvatinib, osimertinib, oxaliplatin, panobinostat, pazopanib, ribociclib, romidepsin, sorafenib, sunitinib, tegafur, toremifene, trifluridine-tipiracil, vandetanib, vemurafenib, vorinostat** |
| Anesthetic/sedative | **chloral hydrate, propofol** |
| Opioids | buprenorphine, **hydrocodone, loperamide,** methadone |
| Bronchodilators (beta -agonists) | **arformoterol, albuterol, formoterol, levalbuterol, indacaterol, olodaterol, salmeterol, terbutaline, vilanterol** |
| Antidiarrheal | **loperamide** |
| Antiemetics | **ondansetron, granisetron, dolasetron,** droperidol |
| Gastrointestinal Promotility | **cisapride, domperidone, metoclopramide** |
| Gonadotropin-releasing hormone agonists and antagonists (GnRH) | **buserelin, degarelix, goserelin, histrelin, leuprolide, triptorelin** |
| Neurologic drugs | **apomorphine, deutetrabenazine, donepezil, ezogabine, fingolimod, pimavanserin, tetrabenazine** |
| Antipsychotics | **chlorpromazine, haloperidol, levosulpiride, methotrimeprazine(levomepromazine), pimozide, sulpiride, thioridazine, amisulpride, aripiprazole, asenapine, clozapine, cyamemazine, flupentixol, iloperidone, melperone, olanzapine, paliperidone, perphenazine, pimavanserin, pipamperone, quetiapine, risperidone, sertindole, tiapride, ziprasidone** |
| Tricyclic and tetracyclic antidepressants (TCAs) | **amitriptyline, amoxapine, clomipramine,** desipramine, **doxepin, imipramine, maprotiline, nortriptyline,** protriptyline, trimipramine |
| Selective serotonin reuptake inhibitors (SSRI) | **citalopram, escitalopram, fluoxetine, fluvoxamine, paroxetine, sertraline** |
| Vasodilator drugs | **bepridil, cilostazol** |
| *Bold indicates the medicine used at the subject hospital | |

| **Table S2. Comorbidities (ICD- 10 code) used in this study** | |
| --- | --- |
| Comorbidities | ICD-10 |
| Myocardial infarction | I21.x, I22.x, I25.2 |
| Congestive heart failure | I09.9, I11.0, I13.0, I13.2, I25.5, I42.0, I42.5–I42.9, I43.x, I50.x, P29.0 |
| Cerebrovascular disease | G45.x, G46.x, H34.0, I60.x–I69.x |
| Ischemic stroke | I63, G45 |
| Hemorrhagic stroke | I60, I61, I62 |
| Diabetes mellitus | E10.x E11.x E12.x E13.x |
| Hypothyroidism | E00.x–E03.x, E89.0 |
| Renal disease | I12.0, I13.1, N03.2–N03.7, N05.2– N05.7, N18.x, N19.x, N25.0, Z49.0– Z49.2, Z94.0, Z99.2 |
| AIDS/HIV | B20.x–B22.x, B24.x |
| Alcohol abuse | F10, E52, G62.1, I42.6, K29.2, K70.0, K70.3, K70.9, T51.x, Z50.2, Z71.4, Z72.1 |
| Drug abuse | F11.x–F16.x, F18.x, F19.x, Z71.5, Z72.2 |
| Liver disease | B18.x, K70.0–K70.3, K70.9, K71.3–K71.5, K71.7, K73.x, K74.x, K76.0, K76.2–K76.4, K76.8, K76.9, Z94.4 |
| Severe liver disease | I85.0, I85.9, I86.4, I98.2, K70.4, K71.1, K72.1, K72.9, K76.5, K76.6, K76.7 |

**Table S3. Detailed baseline characteristics of the subjects in comparison 1 (QT prolongation)**

|  | Total | | QT prolongation | | Control | | p-value |
| --- | --- | --- | --- | --- | --- | --- | --- |
| Total, n (%) | 402607 | 100.0 | 62007 | 15.40 | 340600 | 84.60 |  |
| Sex, n (%) |  |  |  |  |  |  | <.0001 |
| Men | 207742 | 51.6 | 33112 | 53.4 | 174630 | 51.3 |  |
| Women | 194865 | 48.4 | 28895 | 46.6 | 165970 | 48.7 |  |
| Age, mean (SD) | 49.47 | 19.83 | 57.96 | 18.33 | 47.92 | 19.71 | <.0001 |
| Age, n (%) |  |  |  |  |  |  | <.0001 |
| -29 | 67346 | 16.7 | 4693 | 7.6 | 62653 | 18.4 |  |
| 30 - 39 | 50898 | 12.6 | 4468 | 7.2 | 46430 | 13.6 |  |
| 40 - 49 | 69076 | 17.2 | 8244 | 13.3 | 60832 | 17.9 |  |
| 50 - 59 | 74695 | 18.6 | 11593 | 18.7 | 63102 | 18.5 |  |
| 60 - 69 | 72249 | 17.9 | 14088 | 22.7 | 58161 | 17.1 |  |
| 70 - 79 | 53634 | 13.3 | 13966 | 22.5 | 39668 | 11.6 |  |
| 80 - | 14709 | 3.7 | 4955 | 8.0 | 9754 | 2.9 |  |
| Potassium (mEq/l), mean (SD) | 4.16 | 0.49 | 4.09 | 0.62 | 4.17 | 0.46 | <.0001 |
| Calcium (mg/dl), mean (SD) | 9.06 | 0.65 | 8.80 | 0.81 | 9.10 | 0.61 | <.0001 |
| Azithromycin, n (%) | 1305 | 0.32 | 402 | 0.65 | 903 | 0.27 | <.0001 |
| Amoxicilin, n (%) | 5484 | 1.36 | 733 | 1.18 | 4751 | 1.39 | <.0001 |
| Antiarrhythmic drugs, n (%) | 3690 | 0.92 | 1638 | 2.64 | 2052 | 0.60 | <.0001 |
| Antianginal drugs, n (%) | 15 | 0.00 | 6 | 0.01 | 9 | 0.00 | 0.019 |
| Anticholinergic, n (%) | 632 | 0.16 | 159 | 0.26 | 473 | 0.14 | <.0001 |
| Antimallarials, n (%) | 1439 | 0.36 | 238 | 0.38 | 1201 | 0.35 | 0.231 |
| Antifungals, n (%) | 1047 | 0.26 | 239 | 0.39 | 808 | 0.24 | <.0001 |
| Fluoroquinolone antibiotics, n (%) | 13116 | 3.26 | 2799 | 4.51 | 10317 | 3.03 | <.0001 |
| HIV antiretrovirals, n (%) | 51 | 0.01 | 12 | 0.02 | 39 | 0.01 | 0.108 |
| Marcodile antibiotics, n (%) | 7220 | 1.79 | 1817 | 2.93 | 5403 | 1.59 | <.0001 |
| Antihistamines, n (%) | 1626 | 0.40 | 374 | 0.60 | 1252 | 0.37 | <.0001 |
| Antineoplastic drugs, n (%) | 3847 | 0.96 | 543 | 0.88 | 3304 | 0.97 | 0.026 |
| Anesthetic/sedative, n (%) | 11901 | 2.96 | 2060 | 3.32 | 9841 | 2.89 | <.0001 |
| Opioids, n (%) | 2168 | 0.54 | 521 | 0.84 | 1647 | 0.48 | <.0001 |
| Bronchodilators (beta -agonists) , n (%) | 3463 | 0.86 | 713 | 1.15 | 2750 | 0.81 | <.0001 |
| Antidiarrheal, n (%) | 1990 | 0.49 | 485 | 0.78 | 1505 | 0.44 | <.0001 |
| Antiemetics, n (%) | 2611 | 0.65 | 543 | 0.88 | 2068 | 0.61 | <.0001 |
| Promotility, n (%) | 36034 | 8.95 | 9357 | 15.09 | 26677 | 7.83 | <.0001 |
| GnRH, n (%) | 163 | 0.04 | 36 | 0.06 | 127 | 0.04 | <.0001 |
| Neurologic drugs, n (%) | 425 | 0.11 | 147 | 0.24 | 278 | 0.08 | <.0001 |
| Antipsychotics, n (%) | 10199 | 2.53 | 2280 | 3.68 | 7919 | 2.33 | <.0001 |
| Tricyclic and tetracyclic antidepressants (TCAs), n (%) | 4482 | 1.11 | 685 | 1.10 | 3797 | 1.11 | 0.826 |
| Selective serotonin reuptake inhibitors (SSRI), n (%) | 2484 | 0.62 | 483 | 0.78 | 2001 | 0.59 | <.0001 |
| Vasodilator drugs, n (%) | 2749 | 0.68 | 861 | 1.39 | 1888 | 0.55 | <.0001 |
| Myocardial infarction, n (%) | 9886 | 2.46 | 2559 | 4.13 | 7327 | 2.15 | <.0001 |
| Congestive heart failure, n (%) | 8125 | 2.02 | 3284 | 5.30 | 4841 | 1.42 | <.0001 |
| Ischemic stroke, n (%) | 12208 | 3.03 | 3409 | 5.50 | 8799 | 2.58 | <.0001 |
| Hemorrhagic stroke, n (%) | 4142 | 1.03 | 1358 | 2.19 | 2784 | 0.82 | <.0001 |
| Diabetes mellitus, n (%) | 37843 | 9.40 | 8078 | 13.03 | 29765 | 8.74 | <.0001 |
| Hypothyroidism, n (%) | 3363 | 0.84 | 551 | 0.89 | 2812 | 0.83 | 0.113 |
| Renal disease, n (%) | 13262 | 3.29 | 4935 | 7.96 | 8327 | 2.44 | <.0001 |
| AIDS/HIV, n (%) | 169 | 0.04 | 34 | 0.05 | 135 | 0.04 | 0.089 |
| Alcohol abuse, n (%) | 3202 | 0.80 | 1351 | 2.18 | 1851 | 0.54 | <.0001 |
| Drug abuse, n (%) | 695 | 0.17 | 214 | 0.35 | 481 | 0.14 | <.0001 |
| Liver disease, n (%) | 6725 | 1.67 | 2753 | 4.44 | 3972 | 1.17 | <.0001 |
| Severe liver disease, n (%) | 1577 | 0.39 | 818 | 1.32 | 759 | 0.22 | <.0001 |
| Year of QTc diagnosis, n (%) |  |  |  |  |  |  | <.0001 |
| 1995-2000 | 13201 | 3.28 | 1585 | 2.56 | 11616 | 3.41 |  |
| 2001-2005 | 103991 | 25.83 | 11385 | 18.36 | 92606 | 27.19 |  |
| 2006-2010 | 134661 | 33.45 | 20799 | 33.54 | 113862 | 33.43 |  |
| 2011-2015 | 150754 | 37.44 | 28238 | 45.54 | 122516 | 35.97 |  |

**Table S4. Detailed baseline characteristics of the subjects in comparison 2 (Severe QT prolongation)**

|  | Total | | Severe QT prolongation | | Control | | p-value |
| --- | --- | --- | --- | --- | --- | --- | --- |
| Total, n (%) | 402607 | 100.0 | 9353 | 2.32 | 393254 | 97.68 |  |
| Sex, n (%) |  |  |  |  |  |  | <.0001 |
| Men | 207742 | 51.6 | 4519 | 48.3 | 203223 | 51.7 |  |
| Women | 194865 | 48.4 | 4834 | 51.7 | 190031 | 48.3 |  |
| Age, mean (SD) | 49.5 | 19.8 | 61.2 | 17.5 | 49.2 | 19.8 | <.0001 |
| Age, n (%) |  |  |  |  |  |  | <.0001 |
| -29 | 67346 | 16.7 | 513 | 5.5 | 66833 | 17.0 |  |
| 30 - 39 | 50898 | 12.6 | 536 | 5.7 | 50362 | 12.8 |  |
| 40 - 49 | 69076 | 17.2 | 1092 | 11.7 | 67984 | 17.3 |  |
| 50 - 59 | 74695 | 18.6 | 1522 | 16.3 | 73173 | 18.6 |  |
| 60 - 69 | 72249 | 17.9 | 2126 | 22.7 | 70123 | 17.8 |  |
| 70 - 79 | 53634 | 13.3 | 2482 | 26.5 | 51152 | 13.0 |  |
| 80 - | 14709 | 3.7 | 1082 | 11.6 | 13627 | 3.5 |  |
| Potassium (mEq/l), mean (SD) | 4.16 | 0.49 | 4.00 | 0.75 | 4.16 | 0.48 | <.0001 |
| Calcium (mg/dl), mean (SD) | 9.06 | 0.65 | 8.58 | 1.02 | 9.07 | 0.64 | <.0001 |
| Azithromycin, n (%) | 1305 | 0.32 | 83 | 0.89 | 1222 | 0.31 | <.0001 |
| Amoxicilin, n (%) | 5484 | 1.36 | 89 | 0.95 | 5395 | 1.37 | 0.001 |
| Antiarrhythmic drugs, n (%) | 3690 | 0.92 | 569 | 6.08 | 3121 | 0.79 | <.0001 |
| Antianginal drugs, n (%) | 15 | 0.00 | 0 | 0.00 | 15 | 0.00 | 1.000 |
| Anticholinergic, n (%) | 632 | 0.16 | 21 | 0.22 | 611 | 0.16 | 0.095 |
| Antimallarials, n (%) | 1439 | 0.36 | 27 | 0.29 | 1412 | 0.36 | 0.260 |
| Antifungals, n (%) | 1047 | 0.26 | 46 | 0.49 | 1001 | 0.25 | <.0001 |
| Fluoroquinolone antibiotics, n (%) | 13116 | 3.26 | 488 | 5.22 | 12628 | 3.21 | <.0001 |
| HIV antiretrovirals, n (%) | 51 | 0.01 | 0 | 0.00 | 51 | 0.01 | 0.271 |
| Marcodile antibiotics, n (%) | 7220 | 1.79 | 357 | 3.82 | 6863 | 1.75 | <.0001 |
| Antihistamines, n (%) | 1626 | 0.40 | 61 | 0.65 | 1565 | 0.40 | 0.000 |
| Antineoplastic drugs, n (%) | 3847 | 0.96 | 41 | 0.44 | 3806 | 0.97 | <.0001 |
| Anesthetic/sedative, n (%) | 11901 | 2.96 | 335 | 3.58 | 11566 | 2.94 | 0.000 |
| Opioids, n (%) | 2168 | 0.54 | 78 | 0.83 | 2090 | 0.53 | <.0001 |
| Bronchodilators (beta -agonists) , n (%) | 3463 | 0.86 | 100 | 1.07 | 3363 | 0.86 | 0.027 |
| Antidiarrheal, n (%) | 1990 | 0.49 | 74 | 0.79 | 1916 | 0.49 | <.0001 |
| Antiemetics, n (%) | 2611 | 0.65 | 80 | 0.86 | 2531 | 0.64 | 0.012 |
| Promotility, n (%) | 36034 | 8.95 | 1538 | 16.44 | 34496 | 8.77 | <.0001 |
| GnRH, n (%) | 163 | 0.04 | 2 | 0.02 | 161 | 0.04 | 0.353 |
| Neurologic drugs, n (%) | 425 | 0.11 | 33 | 0.35 | 392 | 0.10 | <.0001 |
| Antipsychotics, n (%) | 10199 | 2.53 | 379 | 4.05 | 9820 | 2.50 | <.0001 |
| Tricyclic and tetracyclic antidepressants (TCAs), n (%) | 4482 | 1.11 | 86 | 0.92 | 4396 | 1.12 | 0.071 |
| Selective serotonin reuptake inhibitors (SSRI), n (%) | 2484 | 0.62 | 80 | 0.86 | 2404 | 0.61 | 0.003 |
| Vasodilator drugs, n (%) | 2749 | 0.68 | 160 | 1.71 | 2589 | 0.66 | <.0001 |
| Myocardial infarction, n (%) | 9886 | 2.46 | 551 | 5.89 | 9335 | 2.37 | <.0001 |
| Congestive heart failure, n (%) | 8125 | 2.02 | 980 | 10.48 | 7145 | 1.82 | <.0001 |
| Ischemic stroke, n (%) | 12208 | 3.03 | 617 | 6.60 | 11591 | 2.95 | <.0001 |
| Hemorrhagic stroke, n (%) | 4142 | 1.03 | 359 | 3.84 | 3783 | 0.96 | <.0001 |
| Diabetes mellitus, n (%) | 37843 | 9.40 | 1239 | 13.25 | 36604 | 9.31 | <.0001 |
| Hypothyroidism, n (%) | 3363 | 0.84 | 96 | 1.03 | 3267 | 0.83 | 0.040 |
| Renal disease, n (%) | 13262 | 3.29 | 1050 | 11.23 | 12212 | 3.11 | <.0001 |
| AIDS/HIV, n (%) | 169 | 0.04 | 2 | 0.02 | 167 | 0.04 | 0.325 |
| Alcohol abuse, n (%) | 3202 | 0.80 | 302 | 3.23 | 2900 | 0.74 | <.0001 |
| Drug abuse, n (%) | 695 | 0.17 | 56 | 0.60 | 639 | 0.16 | <.0001 |
| Liver disease, n (%) | 6725 | 1.67 | 614 | 6.56 | 6111 | 1.55 | <.0001 |
| Severe liver disease, n (%) | 1577 | 0.39 | 205 | 2.19 | 1372 | 0.35 | <.0001 |
| Year of QTc diagnosis, n (%) |  |  |  |  |  |  | <.0001 |
| 1995-2000 | 13201 | 3.28 | 314 | 3.36 | 12887 | 3.28 |  |
| 2001-2005 | 103991 | 25.83 | 2288 | 24.46 | 101703 | 25.86 |  |
| 2006-2010 | 134661 | 33.45 | 2976 | 31.82 | 131685 | 33.49 |  |
| 2011-2015 | 150754 | 37.44 | 3775 | 40.36 | 146979 | 37.38 |  |
